# Instructional leadership and teacher job satisfaction as psychological resources for social sustainability: evidence from secondary schools in Northern Cyprus

**DOI:** 10.3389/fpsyg.2026.1948614

**Published:** 2026-10-26

**Authors:** Güliz Başaraner

**Affiliations:** 1 Ministry of Education and Culture (Cyprus), Akropoli, Cyprus; 2 Kibris Aydin Universitesi, Ozanköy, Cyprus

**Keywords:** Northern Cyprus, school leadership, secondary education, shared instructional leadership, social sustainability, teacher job satisfaction, teacher wellbeing

## Abstract

Teacher wellbeing has become an increasingly important concern in sustainable education systems because teachers’ professional experiences influence instructional quality, organizational resilience, and the long-term capacity of schools to maintain supportive learning environments. Contemporary perspectives on educational sustainability emphasize that schools are sustainable not only through academic effectiveness but also through their ability to foster participation, professional recognition, supportive relationships, and psychological resources among teachers. Within this perspective, shared instructional leadership can be considered an organizational resource, while teacher job satisfaction represents an important psychological resource contributing to the human foundations of socially sustainable schools. This study examined the relationship between shared instructional leadership and teacher job satisfaction among secondary school teachers in Northern Cyprus. A quantitative correlational research design was employed with 300 teachers working in public secondary schools during the 2024–2025 academic year. Data were collected using the Shared Instructional Leadership Scale and the Teacher Job Satisfaction Scale. Descriptive statistics, Pearson correlation analysis, and simple linear regression analysis were conducted to examine the relationship between shared instructional leadership and teacher job satisfaction. The findings revealed a significant positive relationship between shared instructional leadership and overall teacher job satisfaction (*r* = 0.580, *p* < 0.001). Within this perspective, shared instructional leadership is conceptualized as an organizational resource, while teacher job satisfaction represents a psychological indicator of teachers’ positive professional experiences. (*β* = 0.580, *p* < 0.001), explaining 33.4% of the variance in teacher job satisfaction (*R*^2^ = 0.334). Significant positive relationships were also found between shared instructional leadership and several dimensions of job satisfaction, including management, achievement–respect–recognition, interpersonal relationships, and the nature of teaching work. The findings suggest that shared instructional leadership may contribute to socially sustainable schooling by strengthening teachers’ professional recognition, relational experiences, and psychological resources. Although social sustainability was not directly measured in this study, the findings highlight leadership practices and teacher satisfaction as important organizational and psychological conditions supporting sustainable educational environments. This study contributes to educational sustainability and leadership literature by emphasizing the human dimension of sustainable schools within the underrepresented context of Northern Cyprus.

## 1 Introduction

### 1.1 Education, sustainability, and the human dimension of schools

Sustainability in education has expanded beyond its initial emphasis on environmental responsibility and resource management to include broader social dimensions related to equity, participation, wellbeing, inclusion, and the quality of relationships within educational organizations (Boström, 2012; Dempsey et al., 2011; Sterling, 2010; Tilbury, 2011; UNESCO, 2020). This broader understanding recognizes that sustainable educational systems depend not only on measurable academic outcomes but also on their ability to maintain supportive environments in which individuals can participate meaningfully, develop professionally, and sustain their commitment over time (UNESCO, 2020).

Schools are fundamentally human organizations. Their long-term capacity for improvement depends largely on the experiences, motivation, and professional engagement of teachers. When teachers experience supportive leadership, recognition, participation in decision-making, and positive professional relationships, schools are more likely to develop cultures characterized by trust, collaboration, and collective responsibility. Conversely, environments where teachers experience limited support or reduced professional satisfaction may weaken organizational stability and hinder sustainable development.

From this perspective, social sustainability represents an important dimension of educational sustainability because it focuses on maintaining the human and relational conditions necessary for organizations to function effectively over time. Socially sustainable schools are not defined solely by institutional continuity or academic achievement; rather, they are characterized by their ability to support the people who sustain educational processes. Teacher wellbeing, professional belonging, recognition, and workplace relationships therefore represent important foundations of sustainable educational environments (Day et al., 2009; Fullan, 2005; Hargreaves and Fink, 2006; OECD, 2019; Viac and Fraser, 2020).

Although social sustainability has gained increasing attention in educational discussions, empirical research examining the organizational mechanisms that contribute to its human dimension remains limited. In particular, further research is needed to understand how leadership practices influence teachers’ professional experiences and how these experiences contribute to sustainable school cultures. However, existing evidence has predominantly emerged from Western educational systems, leaving smaller and less internationally represented contexts insufficiently explored. Examining Northern Cyprus provides an opportunity to understand how leadership practices operate within a distinctive educational setting characterized by specific institutional and cultural conditions.

### 1.2 Shared instructional leadership as an organizational resource for sustainable schools

School leadership plays a central role in shaping the conditions under which teachers work, collaborate, and develop professionally (Day et al., 2009; Hallinger, 2003, 2011; Robinson et al., 2008). Traditional perspectives of school leadership have often emphasized the role of principals as individual decision-makers; however, contemporary approaches increasingly highlight collaborative and distributed forms of leadership in which instructional improvement becomes a collective responsibility (Fullan, 2005; Gümüş et al., 2018; Hallinger and Heck, 2010; Harris, 2020; Spillane, 2006).

Shared instructional leadership reflects this shift by emphasizing collaboration, teacher participation, collective responsibility, and shared engagement in instructional processes. Rather than positioning teachers as passive recipients of administrative decisions, shared instructional leadership recognizes teachers as active contributors to school improvement and instructional development.

This perspective aligns closely with the principles of social sustainability. Participation, recognition, and relational trust are central elements of sustainable organizations because they strengthen individuals’ sense of belonging and collective responsibility. When teachers perceive that their expertise is valued and that they have meaningful opportunities to contribute to instructional decisions, they may develop stronger professional commitment and more positive workplace experiences.

Previous research has demonstrated that instructional leadership is associated with teacher motivation, professional commitment, self-efficacy, and job satisfaction. However, much of the existing literature has focused primarily on school effectiveness and student achievement outcomes. Less attention has been given to how leadership practices contribute to the human dimension of sustainability by supporting teachers’ psychological resources and professional wellbeing.

### 1.3 Teacher job satisfaction as a psychological resource

Teacher job satisfaction is a multidimensional construct reflecting teachers’ evaluations of their professional roles, working conditions, relationships, recognition, and sense of accomplishment (Dinham and Scott, 2000; Herzberg et al., 1959; Klassen and Chiu, 2010). High levels of job satisfaction are associated with positive professional outcomes, including greater motivation, organizational commitment, and willingness to engage in school improvement efforts (Collie et al., 2016; Ingersoll, 2001; Skaalvik and Skaalvik, 2011, 2017; Wang et al., 2022).

Within the social sustainability perspective, teacher job satisfaction can be understood as an important psychological resource rather than as social sustainability itself. Teachers who experience satisfaction in their professional lives are more likely to maintain positive engagement with their schools and contribute to stable and supportive organizational cultures.

Self-determination theory suggests that individuals experience greater motivation and wellbeing when their psychological needs for autonomy, competence, and relatedness are supported (Deci and Ryan, 2000; Aelterman et al., 2017; Ryan and Deci, 2017). Leadership practices that encourage participation, provide recognition, and strengthen professional relationships may therefore contribute to teacher satisfaction by supporting these fundamental psychological needs.

Accordingly, shared instructional leadership may influence teacher job satisfaction by creating conditions where teachers experience professional value, autonomy, and connection. These experiences represent important human-centered conditions that support socially sustainable educational environments.

### 1.4 The present study

Despite increasing recognition of the importance of sustainability in education, limited empirical research has examined how leadership practices contribute to the human and relational dimensions of sustainable schooling. Existing research has generally examined instructional leadership in relation to academic effectiveness, while the connection between leadership, teacher satisfaction, and social sustainability remains underexplored.

This study addresses this gap by examining the relationship between shared instructional leadership and teacher job satisfaction among secondary school teachers in Northern Cyprus. The study conceptualizes shared instructional leadership as an organizational resource and teacher job satisfaction as a psychological resource that contributes to the conditions supporting socially sustainable schools.

The study does not examine social sustainability as a directly measured outcome. Instead, it investigates two important organizational and psychological dimensions that represent the human foundations of sustainable educational environments.

Accordingly, the following research question guides the study:

*RQ1*: To what extent does shared instructional leadership predict teacher job satisfaction among secondary school teachers in Northern Cyprus?

By addressing this question, the study contributes to educational leadership and sustainability literature by demonstrating how leadership practices may support the professional wellbeing and relational capacity necessary for sustainable schools.

## 2 Theoretical framework and research model

### 2.1 Social sustainability perspective in educational organizations

This study is grounded in the social sustainability perspective, which emphasizes the importance of human relationships, participation, inclusion, wellbeing, and organizational capacity in maintaining sustainable institutions (Boström, 2012; Sterling, 2010). Within educational contexts, sustainability involves not only maintaining academic performance but also ensuring that schools possess the social and psychological capacity required for long-term development.

Schools depend heavily on teachers’ professional engagement, motivation, and commitment. Therefore, sustaining educational systems requires attention to the conditions that support teachers’ experiences within their workplaces. Leadership practices that promote participation, recognition, collaboration, and professional empowerment may contribute to the development of school environments where teachers are more likely to experience satisfaction and remain engaged.

In this study, social sustainability is not conceptualized as a directly measured variable. Rather, it provides the theoretical framework through which shared instructional leadership and teacher job satisfaction are examined. Shared instructional leadership is conceptualized as an organizational resource that shapes teachers’ professional experiences, while teacher job satisfaction is conceptualized as a psychological resource reflecting positive workplace experiences.

### 2.2 Shared instructional leadership and teacher job satisfaction

The relationship between leadership practices and teacher outcomes has been widely examined in educational leadership research. Instructional leadership has traditionally focused on principals’ responsibility for improving teaching and learning; however, contemporary approaches emphasize more collaborative forms of leadership in which instructional improvement is developed collectively among school members.

Shared instructional leadership reflects this collaborative orientation by distributing instructional responsibility among school leaders and teachers. Through shared decision-making, professional dialogue, and collective engagement, this leadership approach may strengthen teachers’ perceptions of autonomy, recognition, and professional belonging.

These processes are consistent with self-determination theory, which proposes that individuals experience greater motivation and wellbeing when their needs for autonomy, competence, and relatedness are supported (Deci and Ryan, 2000). In school contexts, leadership practices that encourage teacher involvement and professional recognition may therefore enhance job satisfaction.

Previous empirical studies have demonstrated positive relationships between instructional leadership and teacher job satisfaction. However, examining this relationship through the lens of social sustainability provides a broader understanding of why leadership matters: leadership influences not only instructional processes but also the psychological and relational conditions that sustain educational organizations.

Based on this theoretical position, the proposed research model is presented as follows:

Shared Instructional Leadership → Teacher Job Satisfaction → Conditions Supporting Socially Sustainable Schools.

Accordingly, the following hypothesis was developed:

*H1*: Shared instructional leadership significantly predicts teacher job satisfaction among secondary school teachers.

## 3 Methods

### 3.1 Research design

This study employed a quantitative correlational research design to examine the relationship between shared instructional leadership and teacher job satisfaction among secondary school teachers in Northern Cyprus.

A correlational design was selected because the purpose of the study was to investigate the association between teachers’ perceptions of shared instructional leadership and their levels of job satisfaction, as well as to determine the predictive contribution of shared instructional leadership to teacher job satisfaction. The study was theoretically informed by the social sustainability perspective. Within this framework, shared instructional leadership was examined as an organizational resource and teacher job satisfaction as a psychological resource contributing to the human foundations of socially sustainable schools. Although social sustainability itself was not directly measured, the study examined two important conditions through which sustainable educational environments may be supported: collaborative leadership practices and positive teacher professional experiences.

### 3.2 Participants and research context

The population of the study consisted of approximately 1,400 teachers working in 20 public secondary schools located in the Nicosia and Kyrenia districts of Northern Cyprus during the 2024–2025 academic year. A total of 300 teachers participated in the study through a random sampling procedure. The sample size was considered adequate for the statistical analyses conducted. Among the participants, 67% were female and 33% were male. Regarding marital status, 62.67% of teachers were married. In terms of educational background, 53.67% were graduates of faculties of education. Participants represented different age groups and professional experience levels, allowing examination of teachers’ perceptions across diverse professional backgrounds.

### 3.3 Data collection procedure and ethical considerations

Data were collected during the 2024–2025 academic year following institutional permission procedures. Participation was voluntary, and participants were informed about the purpose of the study before completing the questionnaires. Confidentiality and anonymity were maintained throughout the research process. Participants were not required to provide identifying information, and the collected data were used solely for research purposes. Ethical principles regarding voluntary participation, informed consent, and confidentiality were followed throughout the study.

### 3.4 Data collection instruments

#### 3.4.1 Shared Instructional Leadership Scale

Shared instructional leadership was measured using the Shared Instructional Leadership Scale (SILS) developed by Zhan et al. (2020). The scale consists of seven items rated on a six-point Likert scale. The instrument evaluates teachers’ perceptions of collaborative instructional leadership practices, including shared responsibility, participation in instructional decisions, and collective engagement in improving teaching practices. In the present study, the Cronbach’s alpha reliability coefficient of the scale was calculated as α = 0.763, indicating acceptable internal consistency. Within the theoretical framework of this study, shared instructional leadership represents an organizational resource because participatory leadership practices may strengthen teachers’ professional agency, recognition, and sense of belonging (Nunnally and Bernstein, 1994).

#### 3.4.2 Teacher job satisfaction scale

Teacher job satisfaction was measured using the Teacher Job Satisfaction Scale developed by Şahin (1999). The scale includes 42 items rated on a three-point Likert scale (“Yes,” “Partially,” and “No”). The instrument consists of six dimensions:

- Management
- Salary
- Achievement–Respect–Recognition
- Interpersonal Relationships
- Work Itself
- Parent–Student Indifference

In the present study, the Cronbach’s alpha reliability coefficient was α = 0.732, demonstrating acceptable internal consistency. Teacher job satisfaction was conceptualized as a psychological resource reflecting teachers’ evaluations of their professional experiences, including recognition, relationships, and satisfaction with their work environment.

### 3.5 Data analysis

Data analysis was conducted using descriptive statistics, Pearson product–moment correlation analysis, and simple linear regression analysis. First, descriptive statistics were calculated to determine teachers’ perceptions of shared instructional leadership and teacher job satisfaction. Second, Pearson correlation analysis was conducted to examine the relationship between shared instructional leadership and overall as well as dimensional measures of teacher job satisfaction. Third, simple linear regression analysis was performed to determine whether shared instructional leadership significantly predicted teacher job satisfaction. Statistical significance was evaluated at the *p* < 0.05 level.

The analytical approach was aligned with the theoretical framework by examining how organizational leadership practices relate to teachers’ psychological experiences, which represent important conditions supporting socially sustainable educational environments.

## 4 Results

### 4.1 Descriptive statistics

Descriptive statistics were calculated to examine teachers’ perceptions of shared instructional leadership and teacher job satisfaction.

The results indicated that teachers reported relatively high perceptions of shared instructional leadership. The overall mean score for shared instructional leadership was *M* = 4.65 (SD = 0.24) on a six-point Likert scale. This finding indicates that teachers generally perceived instructional leadership practices in their schools as involving collaboration, shared responsibility, and collective engagement in instructional processes.

The overall mean score for teacher job satisfaction was *M* = 2.14 (SD = 0.18) on a three-point Likert scale, indicating a moderate level of satisfaction among teachers.

The descriptive statistics for the dimensions of teacher job satisfaction are presented below:

**Table 1:** 

Teacher job satisfaction dimension	Mean (M)	Standard deviation (SD)
Management	2.20	0.30
Salary	1.98	0.39
Achievement–respect–recognition	2.25	0.26
Interpersonal relationships	2.01	0.33
Work itself	2.36	0.31
Parent–student indifference	1.83	0.39
Overall job satisfaction	2.14	0.18

Among the dimensions of teacher job satisfaction, the highest mean score was observed for Work Itself (*M* = 2.36, SD = 0.31), followed by Achievement–Respect–Recognition (*M* = 2.25, SD = 0.26) and Management (*M* = 2.20, SD = 0.30). The lowest mean score was found for Parent–Student Indifference (*M* = 1.83, SD = 0.39), followed by Salary (*M* = 1.98, SD = 0.39).

These results suggest that teachers reported stronger satisfaction regarding intrinsic and professional aspects of teaching, whereas external factors such as salary and parent–student-related experiences represented comparatively lower satisfaction areas.

### 4.2 Relationship between shared instructional leadership and teacher job satisfaction

Pearson product–moment correlation analysis was conducted to examine the relationship between shared instructional leadership and teacher job satisfaction.

The findings demonstrated a statistically significant positive relationship between shared instructional leadership and overall teacher job satisfaction:

*r* = 0.580, *p* < 0.001.

This result indicates that higher perceptions of shared instructional leadership were associated with higher levels of teacher job satisfaction.

The relationships between shared instructional leadership and the dimensions of teacher job satisfaction were also examined. The results are presented below:

**Table 2:** 

Teacher job satisfaction dimension	Correlation with shared instructional leadership
Management	*r* = 0.641, *p* < 0.001
Salary	*r* = 0.415, *p* < 0.001
Achievement–respect–recognition	*r* = 0.636, *p* < 0.001
Interpersonal relationships	*r* = 0.622, *p* < 0.001
Work itself	*r* = 0.615, *p* < 0.001
Parent–student indifference	*r* = 0.042, *p* > 0.05

Significant positive relationships were found between shared instructional leadership and all dimensions of teacher job satisfaction except parent–student indifference.

The strongest associations were observed for:

- Management (*r* = 0.641)
- Achievement–Respect–Recognition (*r* = 0.636)
- Interpersonal Relationships (*r* = 0.622)
- Work Itself (*r* = 0.615)

The relationship between shared instructional leadership and parent–student indifference was not statistically significant (*r* = 0.042, *p* > 0.05).

### 4.3 Predictive effect of shared instructional leadership on teacher job satisfaction

A simple linear regression analysis was conducted to determine whether shared instructional leadership significantly predicted teacher job satisfaction.

The regression model was statistically significant:

*F*(1, 298) = 150.832, *p* < 0.001.

The results indicated that shared instructional leadership significantly predicted teacher job satisfaction:

*β* = 0.580, *p* < 0.001.

The model explained 33.4% of the variance in teacher job satisfaction (*R*^2^ = 0.334).

These findings indicate that shared instructional leadership represents a significant predictor of teachers’ job satisfaction levels. Although teacher job satisfaction is influenced by multiple individual, organizational, and contextual factors, the results demonstrate that leadership practices constitute an important organizational factor associated with teachers’ professional experiences.

#### 4.3.1 Summary of results

Overall, the findings demonstrate that teachers who reported stronger perceptions of shared instructional leadership also reported higher levels of job satisfaction. The significant relationships between leadership and dimensions related to recognition, management, interpersonal relationships, and the nature of teaching work highlight the importance of relational and organizational factors in shaping teachers’ professional experiences.

Within the framework of this study, these findings suggest that shared instructional leadership may represent an important organizational resource supporting the psychological and relational conditions associated with socially sustainable schools.

## 5 Discussion

This study examined the relationship between shared instructional leadership and teacher job satisfaction within the broader perspective of social sustainability in education. The findings demonstrated that shared instructional leadership was positively associated with teacher job satisfaction and significantly predicted teachers’ satisfaction levels. These results suggest that leadership practices based on collaboration, shared responsibility, and teacher participation may function as important organizational resources that support teachers’ professional experiences.

The findings contribute to the growing literature on the human dimension of educational sustainability. Previous approaches to sustainability in education have often emphasized environmental responsibility, institutional effectiveness, or policy-level changes. However, sustainable educational systems also depend on their ability to maintain supportive professional environments where teachers experience recognition, participation, psychological wellbeing, and meaningful professional relationships (Boström, 2012; Sterling, 2010). From this perspective, the present study highlights the importance of leadership practices that support the individuals responsible for sustaining educational processes. Successful school leadership contributes to school improvement by developing organizational conditions that support teachers and collective capacity (Leithwood et al., 2020).

The significant positive relationship between shared instructional leadership and overall teacher job satisfaction is consistent with previous research indicating that collaborative and supportive leadership approaches enhance teachers’ motivation, commitment, and workplace satisfaction (Aelterman et al., 2017; Gümüş et al., 2018; Hallinger and Heck, 2010; Ryan and Deci, 2017). When teachers perceive that their professional knowledge is valued and that they have opportunities to participate in instructional decisions, they may experience stronger feelings of autonomy, competence, and relatedness. These psychological experiences are consistent with self-determination theory, which suggests that supportive social environments contribute to individuals’ motivation and wellbeing (Deci and Ryan, 2000).

The findings also extend instructional leadership research by positioning leadership as more than a mechanism for improving instructional outcomes. Traditionally, instructional leadership has been examined primarily in relation to teaching quality and student achievement. However, the current findings suggest that leadership practices also influence teachers’ professional experiences by shaping workplace relationships, recognition, and participation. These dimensions represent important elements of the social sustainability perspective because sustainable organizations depend on their capacity to maintain positive human relationships and professional commitment over time.

The regression analysis revealed that shared instructional leadership explained 33.4% of the variance in teacher job satisfaction. This finding indicates that leadership practices represent a meaningful organizational factor associated with teachers’ professional satisfaction. However, teacher job satisfaction is a complex construct influenced by multiple personal, organizational, and contextual variables. Therefore, the findings should not be interpreted as suggesting that leadership alone determines teacher satisfaction. Rather, shared instructional leadership represents one important condition that contributes to positive teacher experiences.

The analysis of the dimensions of teacher job satisfaction provides further insight into how leadership practices may influence teachers’ professional lives (Collie et al., 2016; Hargreaves and Fink, 2006; Hargreaves and Fullan, 2012; Skaalvik and Skaalvik, 2011, 2017; Wang et al., 2022). The strongest relationships were observed between shared instructional leadership and management, achievement–respect–recognition, interpersonal relationships, and work itself. These findings suggest that collaborative leadership practices may be particularly important in strengthening teachers’ perceptions of professional value, organizational support, and meaningful work.

The strong association between shared instructional leadership and achievement–respect–recognition is particularly relevant from a social sustainability perspective. Recognition and appreciation are central components of supportive professional environments because they influence teachers’ sense of belonging and organizational identification. Similarly, the relationship between shared instructional leadership and interpersonal relationships highlights the importance of trust and collaboration as foundations of sustainable educational communities.

The non-significant relationship between shared instructional leadership and parent–student indifference provides an additional perspective. This result suggests that some aspects of teacher satisfaction may be influenced by factors beyond the immediate influence of school leadership. Parent involvement, student characteristics, and broader social conditions may shape teachers’ experiences in ways that cannot be fully addressed through leadership practices alone. This finding reinforces the multidimensional nature of teacher job satisfaction and the complexity of creating sustainable educational environments.

The context of Northern Cyprus provides a valuable contribution to the literature. Educational sustainability and leadership research has largely been conducted in larger and more extensively studied educational systems. Evidence from Northern Cyprus contributes to expanding the geographical diversity of research by illustrating how leadership practices operate within a specific cultural and institutional context. The findings suggest that collaborative leadership approaches may support teacher wellbeing and professional engagement even within smaller educational systems.

From a sustainability perspective, the findings indicate that school leaders should consider the development of teacher-centered leadership cultures as an important component of sustainable school development. Leadership practices that encourage participation, professional dialogue, recognition, and shared responsibility may strengthen the psychological and relational resources necessary for schools to maintain continuous improvement.

## 6 Limitations and future research

Several limitations should be considered when interpreting the findings of this study.

First, although the study was theoretically grounded in the social sustainability perspective, social sustainability was not directly measured as a separate construct. Instead, the study examined two important organizational and psychological conditions related to the human foundations of socially sustainable schools: shared instructional leadership as an organizational resource and teacher job satisfaction as a psychological resource. Future research should incorporate direct measures of social sustainability, including organizational trust, teacher retention intentions, professional belonging, collective efficacy, participation, and equity-related indicators.

Second, the study employed a quantitative correlational design based on self-reported data. Although this approach was appropriate for examining relationships among variables, causal interpretations cannot be made. Future studies using longitudinal designs, qualitative approaches, or mixed-method research could provide deeper insights into how leadership practices influence teacher experiences over time. According to Cohen’s (1992) criteria, this represents a medium-to-large practical effect size in educational research contexts.

Third, the study was conducted among secondary school teachers in Northern Cyprus. Although the findings provide valuable evidence from an underrepresented educational context, cultural, institutional, and policy differences may influence the relationship between leadership and teacher satisfaction. Future research involving different educational systems would contribute to broader understanding.

Despite these limitations, the study provides meaningful evidence regarding the role of leadership and teacher experiences in supporting the human dimension of sustainable education.

## 7 Practical implications

The findings provide several implications for school leaders, policymakers, and leadership development programs.

First, school leaders should recognize that instructional leadership is not limited to monitoring teaching practices or improving academic outcomes. Shared instructional leadership can serve as a relational approach that strengthens teacher participation, professional recognition, and collaboration.

Second, schools aiming to develop sustainable organizational cultures should create opportunities for teachers to participate in instructional decision-making processes. Encouraging professional dialogue, collaborative planning, and shared responsibility may enhance teachers’ sense of belonging and psychological wellbeing.

Third, educational policymakers should consider teacher wellbeing as an essential component of sustainable education systems. Policies addressing professional recognition (OECD, 2019; Viac and Fraser, 2020; Wang et al., 2022), supportive working conditions, leadership development, and teacher involvement may contribute to maintaining a motivated and engaged teaching workforce.

Leadership preparation programs may also benefit from emphasizing relational and participatory leadership competencies. Developing principals’ skills in communication, collaboration, trust building, and teacher empowerment may strengthen the organizational conditions necessary for sustainable schools.

## 8 Conclusion

This study examined the relationship between shared instructional leadership and teacher job satisfaction within the framework of social sustainability in education. The findings demonstrated that shared instructional leadership significantly predicted teacher job satisfaction and was positively associated with multiple dimensions of teachers’ professional experiences.

The study contributes to educational sustainability literature by emphasizing that sustainable schools depend not only on academic effectiveness but also on their ability to support the people who sustain educational processes. Teachers’ satisfaction, recognition, participation, and professional relationships represent important human foundations of sustainable educational environments.

Although social sustainability was not directly measured, the findings highlight how leadership practices and teacher experiences may contribute to the development of socially sustainable school conditions. Shared instructional leadership can therefore be understood as an organizational resource that supports teacher wellbeing, professional engagement, and collaborative school cultures.

Future research should continue to explore the connection between educational leadership and social sustainability by incorporating direct sustainability measures and examining how organizational practices influence teachers’ long-term engagement, commitment, and retention.
